# *Vibrio splendidus* virulence to *Apostichopus japonicus* is mediated by hppD through glutamate metabolism and flagellum assembly

**DOI:** 10.1080/21505594.2022.2046949

**Published:** 2022-03-08

**Authors:** Weikang Liang, Weiwei Zhang, Chenghua Li

**Affiliations:** a State Key Laboratory for Managing Biotic and Chemical Threats to the Quality and Safety of Ningbo University, Ningbo, P. R. China; b Laboratory for Marine Fisheries Science and Food Production Processes, Qingdao National Laboratory for Marine Science and Technology, Qingdao, P. R. China

**Keywords:** *Vibrio splendidus*, *Apostichopus japonicus*, 4-Hydroxyphenylpyruvate dioxygenase, Glutamate metabolism, Flagellum assembly

## Abstract

*Vibrio splendidus* is the main opportunistic pathogen that causes skin ulcer syndrome in *Apostichopus japonicus*. *hppD*In the present study, mutant *V. splendidus* with an in-frame deletion of *hppDV.s*. (MTVs) was constructed. The median lethal doses of wild-type *V. splendidus* (WTVs) and MTVs were 5.129 × 10^6^ and 2.606 × 10^10^ CFU mL^−1^, respectively. RNA-Seq was performed using WTVs and MTVs cells at different growth stages to explore the mechanisms of the pathogenesis mediated by hppDV.s. Gene Ontology analysis showed that the expression levels of 105 genes involved in amino acid metabolism and protein binding were remarkably different between MTVs and WTVs. Kyoto Encyclopedia of Genes and Genomes analysis showed that the pathways of glutamate metabolism and flagellum assembly involved in biofilm formation and swarming motility were suppressed in MTVs. Correspondingly, the swarming motility, biofilm formation and colonisation of MTVs were remarkably decreased compared with those of WTVs. The results showed that 4-hppD catalyses tyrosine into fumarate, which could enhance glutamate metabolism and ATP production; promote flagellum assembly through the TCA cycle and lead to higher swarming, biofilm formation and colonisation abilities, to contribute to the pathogenesis of *V. splendidus*.

## Introduction

The *Vibrio* genus causes serious infectious diseases that affect marine animals and humans [1,2]. The bacterium *Vibrio splendidus* naturally inhabits aquatic environments as the dominant vibrioplankton and is also abundant in and on many animal species [3]. Over the past few decades, different *V. splendidus* strains have been demonstrated to be associated with the mortality of cod larvae, turbot and shellfish [4,5]. Sea cucumber *Apostichopus japonicus* (Echinodermata, Holothuroidea) frequently suffer from “skin ulcer syndrome” (SUS) outbreak caused by bacterial infection. The SUSs affecting sea cucumbers probably correspond to different diseases depending on species or geographic location [^6–9^]. The main pathogens include *Vibrio* sp., *Pseudomonas* sp. and even spherical virus; among which, *V. splendidus* was considered the major pathogen that infects *A. japonicus* [10,11]. Compared with human pathogenic *Vibrio* species, little is known about *Vibrio* pathogenesis in aquatic animals. Despite its virulence factors and lethality have been described, few reports have focused on the pathogenic pathway of *V. splendidus* until now.

In the pathogenic process, the production of virulence factors results in metabolism and biological function disorders, which eventually destroy host cells and tissues. The virulence factors of *Vibrio* sp. contain adhesin factor, extracellular metalloprotease, hemolysin, siderophore, lipopolysaccharide and type III secretion system [12]. The main virulence factors of *V. splendidus*, including extracellular metalloprotease Vsm [13,14], quorum sensing [15], the siderophore receptors of LutA and FepA [16] and a hemolysin 4-hydroxyphenylpyruvate dioxygenase (4-hppD) encoded by the *hppD* gene of *V. splendidus* (*hppDV.s*.) [17], have been demonstrated to be related to the fulminant infectious diseases of sea cucumber.

The transformation catalyzed by 4-hppD has vital physiological importance and occurs in almost all aerobic organisms [18]. In aerobic metabolism, 4-hppD catalyses the tyrosine catabolism (that is the transformation of 4-hydroxyphenylpyruvate to homogentisate [HGA]). High HGA concentrations accumulate, self-polymerise and autoxidise into pyomelanin via non-enzymatic reactions; this oxidation process can damage the host and result in a hemolytic phenotype [^18–21^]. Our previous study confirmed the dual functions of hppDV.s. for *V. splendidus* survival and cytotoxicity [22]. On the one hand, hppDV.s. promotes pyomelanin production and provides competitiveness for *V. splendidus* in unfavorable environments [22]. On the other hand, the purified recombinant hppDV.s. possesses cytotoxic effect and hemolytic activity [17]. Thus, pyomelanisation may benefit the survival and perhaps exacerbate the virulence of *Vibrio* sp. under this host environment. However, the regulation of global cellular metabolism and bacterial pathogenicity caused by 4-hppD is not known.

The infectivity and pathogenicity of *Vibrio* sp. are caused by the collective effect of various virulence factors. Recently, the microbial transcriptome sequencing (RNA-seq) technique has dramatically accelerated analytical capacity and has been widely applied to construct global networks of virulence factors [23]. In the present study, a mutant *V. splendidus* with an in-frame deletion of *hppDV.s*. (MTVs) was constructed. The growth curve, hemolytic activity, UV resistance and pathogenicity of wild-type *V. splendidus* (WTVs) and MTVs were analyzed and compared. RNA-seq was used to analyze the RNA from WTVs and MTVs cells collected at different growth stages to reveal the pathogenesis mechanism mediated by *hppDV.s*.

## Materials and methods

### Bacterial strains, culture conditions and chemicals

Pathogenic *V. splendidus* strain was separated from the tissues of sea cucumbers with SUS from the indoor farms of Jinzhou Hatchery in May 2013. The strain’s pathogenicity to *A. japonicus* was demonstrated by reinfection experiment [24]. The strain was identified by 16S rDNA sequencing analysis. *Escherichia coli* DH5α (Takara, China) and S17λ*pir* were cultured in Luria–Bertani broth medium at 37°C with shaking at 200 rpm. *V. splendidus* was cultured in Zobell’s 2216E medium (1 g yeast extract and 5 g tryptone in 1 L seawater, pH 7.6–8.0) at 28°C with shaking at 200 rpm. Ampicillin (Ap, 100 μg mL^−1^), chloramphenicol (Cm, 50 μg mL^−1^) and kanamycin (Kn, 50 μg mL^−1^) were added to the medium when needed. Taq DNA polymerase and pMD19-T vector were purchased from Takara (Beijing, China). Vectors pDM4 and pBT3 were preserved in our laboratory. Restriction endonucleases were purchased from New England Biolabs and used in accordance with the manufacturer’s speciﬁcations.

### MTVs construction and complementation

The plasmid preparation, the extraction of DNA fragments from agarose gels and the puriﬁcation of PCR products were performed using the respective kits from Omega Bio-Tek (GA) according to the manufacturer’s instructions. The primers used in the present research are listed in Table 1. The primers were designed according to the genomic DNA of *V. atlanticus* strain LGP32 with accession number FM954973.2. MTVs was constructed as previously described by Hu et al [25]. The 700 bp upstream fragment and 677 bp downstream fragment of *hppDV.s*. were amplified by polymerase chain reaction (PCR) using KohppDV.s.F1/KohppDV.s.R1 and KohppDV.s.F2/KohppDV.s.R2 and fused by overlap PCR. The 1376 bp fragments were inserted into the pMD19-T vector and then transformed into *E. coli* DH5α. Three positive clones were collected for sequencing at Genewiz Biotechnology (Suzhou, China). The overlap fragment was digested and inserted into the *Bgl* II site of suicide vector pDM4 containing the *sacB* gene to construct plasmid pDM4KohppDV.s.. The plasmid was transformed into S17λ*pir*. The recombinant plasmid pDM4KohppDV.s. was transformed into WTVs by conjugation. Bacterial conjugation was performed as described previously [26]. The conjugants were placed on 2216E agar with Cm and Ap, and the positive conjugants were confirmed by PCR (KohppDV.s.F1/KohppDV.s.R2) and sequencing. The second cross-over recombination was carried out in 2216E agar with 10% sucrose. The MTVs was verified by PCR (KohppDV.s.F1/KohppDV.s.R2) and sequencing.

**Table 1.** Primers used in this study

Primer	Sequences (5′–3′)^a^
KohppDV.s.F1	AGATCTTCATCTCAGCTAGCGTCACTTTTTTAGTAGA (*Bgl* II)
KohppDV.s.R1	CATTTATGCATCGTCTGTATCCACCATGAACTTTCTCCTTGTACC
KohppDV.s.F2	TTCATGGTGGATACAGACGATGCATAAATGGATCTCGTTTCC
KohppDV.s.R2	AGATCTATCCAATGGATTGAACGGATA (*Bgl* II)
8F	AGAGTTTGATCCTGGCTCAG
1492R	GGTTACCTTGTTACGACTT
VsGly1F	ATGGTGGAGTCATACAACCC
VsGly1R	CTCCTCTGCATCATCGTAGA
VsGly2F	TACGATGATGCAGAGGAGCG
VsGly2R	TTATGCATCGTCTAATACTCCTCG
hppD^C^F	GGATCCATGGTGGATACATACAACCCG (*Bam*H I)
hppD^C^R	CTCGAGTTATGCATCGTCTAATACTCCTCGA (*Xho* I)
flgDV.s.RTF	TGTTGAGTGGGACGGAAAAGA
flgDV.s.RTR	CGATGTAAAACCAGCCAGATTG
tpPV.s.RTF	GCGTTTACTCTGCGATTCACC
tpPV.s.RTR	GAAGCCAGACTCCCAACCAA
hsp20V.s.RTF	GGCGGTTACCCTCCATACAA
hsp20V.s.RTR	AAATCACGCTCTGCGATACCT
clpBV.s.RTF	GAGATCGCCGATGTCCTTTC
clpBV.s.RTR	ATACCACTTCGACCGCTTCC
lolBV.s.RTF	TTATGGTGGGTTGCTCGTCTAT
lolBV.s.RTR	GCTTTGATTTGGGGAGTGCTT
hAV.s.RTF	AGATGCTTTTAATACCGTAGGCG
hAV.s.RTR	ACTTGATGTTGTTGGCTTGCTT
933F	GCACAAGCGGTGGAGCATGTGG
16SRTR1	CGTGTGTAGCCCTGGTCGTA

*^a^* Underlined nucleotides are the restriction sites of the enzymes indicated in brackets at the end of the sequences.

Plasmid pET28ahppDV.s. was generated to complement the MTVs with the *hppDV.s*. sequence. The DNA fragment of *hppDV.s*. was amplified by hppD^C^F/hppD^C^R and ligated into pMD19-T for the convenience of subsequent enzyme digestion. Plasmid pBT3 with constitutive promoter P_Trc_ was digested with *Bam*H I and *Xho* I, and plasmid pBT3hppDV.s. was constructed by ligating *hppDV.s*. into pBT3 to reach a higher *hppDV.s*. expression efficiency. The DNA fragment named P_Trc_-fused *hppDV.s*., which contained the Trc promoter and the functional *hppDV.s*. gene, was obtained after pBT3hppDV.s. was digested using *Swa* I. Then, the DNA fragment of the P_Trc_-*hppDV.s*. fragment was inserted into the *Eco*R V site of pET28a, and the recombinant plasmid was transformed into S17λπ to construct S17λπ/pET28ahppDV.s. The recombinant plasmid pET28ahppDV.s. was transformed into MTVs by conjugation. The conjugants were placed on 2216E agar with Kn and Ap, and the positive conjugants were confirmed by PCR and sequencing.

### Growth curve measurement

The WTVs and MTVs were placed in 2216E agar at 28°C overnight. Single colonies were inoculated into tubes with 10 mL of fresh 2216E medium and cultured at 28°C with shaking at 200 rpm. Overnight cultures were diluted to the same concentration, and 200 μL aliquots of WTVs and MTVs were transferred into flasks with 100 mL of fresh 2216E medium. The flasks were incubated at 28°C with shaking at 200 rpm. The optical density at 600 nm (OD_600_) was measured at different time points. Three independent experiments were performed in this manner.

### Hemolytic activity measurement

The hemolytic activity of WTVs and MTVs were measured as described by Liang et al [17]. The same concentrations of WTVs and MTVs cells were spread on 2216E solid medium complemented with 5% sterilised sheep blood and cultured at 28°C to qualitatively detect bacterial hemolytic activity. A hemolytic ring was observed after 24 h. To quantitatively detect hemolytic activity, 10 μL of the strains preserved with glycerol at a ratio of 1:1 (v/v) were inoculated into 10 mL of 2216E medium and incubated at 28°C with shaking at 200 rpm. The WTVs and MTVs cultured to an OD_600_ of 1.0 were centrifuged at 8000 × *g* for 10 min to collect the supernatant, which was then sterile filtered through a 0.22 μm sterile ﬁlter membrane (Millipore, USA). Blood cell preparation was conducted as described by Lee et al [21]. Brieﬂy, cells from sterile deﬁdrinated sheep blood was washed in sterilized Dulbecco’s modiﬁed Eagle medium (DMEM) for three times, and then resuspended in sterilized DMEM to a concentration of 5% (v/v). Equal volume (500 μL) of preheated sheep blood cell suspension was amended with 0, 10, 50 and 100 μL of WTVs and MTVs cell-free supernatants. The 2216E medium served as the control. The released protohemes after incubation at 28°C for 5 h were measured at OD_570_ under a Spectramax 190. Three biological replicates were obtained from the WTVs and MTVs groups.

### Anti-UV activity measurement

The WTVs and MTVs were cultured in 2216E medium at 28°C. The same concentration of bacteria was poured into glass petri plates, which were then exposed to UV light with shaking regularly. Bacterial culture was collected and diluted to approximately 10^4^ CFU mL^−1^ at 0, 5, 10, 15 and 30 min. Then, the dilutions collected at different time points were spread onto the plates. The single colonies of viable WTVs and MTVs were counted after 24 h. The experiment was repeated at least three times.

### Artificial infection

The *A. japonicus* infection experiments were conducted as described by Zhang et al [24]. Healthy sea cucumbers (weight, 3 ± 1 g) were purchased from Shandong Oriental Ocean Company (Yantai, China) and reared in aerated natural seawater (salinity, 28 psu) at 16°C for 3 days. Then, the sea cucumbers were randomly divided into 13 groups with 20 individuals in each group. WTVs and MTVs strains were cultured to an OD_600_ of 1.0 in 2216E medium (28°C 24 h), centrifuged at 8000 × *g*, washed with PBS (28 °C) and resuspended with seawater. For artificial infection, weight-matched *A. japonicus* were continuously immersed with 5 × 10^7^, 1 × 10^7^, 5 × 10^6^, 1 × 10^6^, 5 × 10^5^ and 1 × 10^5^ CFU mL^−1^*V. splendidus* (WTVs or MTVs). *A. japonicus* infected with PBS served as the negative control. The daily mortality of infected *A. japonicus* was recorded. Dead *A. japonicus* were picked out immediately, and the observed symptoms were recorded.

### cDNA library preparation, Illumina sequencing and mapping of reads

For RNA-Seq, the WTVs and MTVs strains were cultured in 2216E agar plates for 24 h. Single colonies of both strains were inoculated into flasks with fresh 2216E medium and cultured at 28°C with shaking at 200 rpm. The WTVs and MTVs cells were collected when OD_600_ reached 0.6, 1.0 and 1.5.

rRNA was removed using the Ribo-zero Kit to enrich the mRNA. Subsequently, mRNA was broken into short fragments using a fragmentation reagent. First-strand cDNA was synthesised using a random hexamer primer and using mRNA as a template. Buffers, DNA polymerase I, RNase H and dNTPs were added to synthesise the double-stranded cDNA. The resulting cDNAs were purified using AMPure XP beads to obtain the final library. Sequencing was conducted on the Illumina HiSeq/MiSeq sequencing platform at Novogene Biotech (Beijing, China). The raw data were submitted to the NCBI Sequence Read Archive (SRA) database.

The trimming and quality control of raw Illumina reads were conducted to obtain clean reads, which were mapped to the genome of *V. atlanticus* strain LGP32 (FM954973.2) using Bowtie2 [27].

### Analysis of differential gene expression

The reads were aligned to the bacterial genome (NCBI FM954973.2) for *V. splendidus* transcriptome analysis. Transcript abundance quantiﬁcation (in fragments per kilobase of transcript sequence per millions base pairs sequenced) was performed using RNA-seq by expectation maximisation with default parameters. Differential expression was determined using the following thresholds: q < 0.005 and |log2^fold change^| ≥ 1.

### Quantification of mRNA expression

The employed specific primers are listed in Table 1. Melting curve analysis and agarose gel electrophoresis were conducted to ensure the specificity of the PCR products. The total RNA of *V. splendidus* was collected from WTVs and MTVs cells with different OD_600_ values. The primers used for the reference control of 16S rDNA were 933F and 16SRTR1. Real-time polymerase chain reaction (RT-PCR) was carried out in an ABI 7500 RT-PCR detection system (Applied Biosystems) with a total volume of 20 μL (8 μL of cDNA, 0.8 μL of each primer [10 mM], 10 μL of TB GREEN^TM^ Premix Ex Taq II and 0.4 μL of Dye-II [ROX]), followed by 40 cycles of 95°C for 15 s, 60°C for 20 s, 72°C for 20 s. The 2^−ΔΔCT^ method was used to analyze the relative expression level of the candidate genes [28].

### Bioﬁlm assay

The bioﬁlm assay for WTVs, MTVs and *hppD^C^* was conducted as described by Luo et al [29]. WTVs, MTVs and *hppD^C^* strains were grown overnight at 28°C in 2216E medium. A 2 μL of bacterial culture was added to a 96-well plate with 198 μL of 2216E medium per well and incubated at 28°C until OD_600_ reached 1.0. Then, the plate was washed three times with sterile PBS, dyed with 200 μL of crystal violet (1%) for 30 min, then washed with sterile PBS and air-dried. The attached bioﬁlm was dissolved with 200 μL of ethanol and then quantified at OD_570_. Five independent biological replicates were conducted.

### Motility analysis

Swarming motility assay was set up as previously described by Wang et al [30]. WTVs, MTVs and *hppD^C^* strains were separately cultured overnight and diluted to 1:100 in fresh 2216E broth. Then, 2 µL of diluted bacterial cultures (OD_600_ = 1.0) were dropped on 2216E plates containing 0.3% agar at 28°C for 12 h to observe the colony size. All the experiments were repeated three times.

### Distribution of *V.* *splendidus* in *A.* *japonicus* tissues

The distribution of *V. splendidus* in the different *A. japonicus* tissues was determined as described previously [31,32]. Six *A. japonicus* individuals were equally divided into two tanks, and each tank was supplemented with 10 L of aerated natural seawater. A total of 0.1 L MTVs (OD_600_ = 1.0) were centrifuged at 8000 × *g* for 10 min and used to infect *A. japonicus*. The control group was *A. japonicus* infected with WTVs (0.1 L, OD_600_ = 1.0). After *A. japonicus* was subjected to immersion infection for 24 h, the tentacle, muscle and body wall of *A. japonicus* were collected using sterilised scissors and tweezers. Three specimens were collected for each *A. japonicus* tissue. The tissue samples were homogenised by a homogeniser and spread on 2216E medium supplemented with Ap (100 μg mL^−1^). No colony other than *V. splendidus* grew on the plate supplemented with Ap. Furthermore, three clones from each plate were identified by 16S rDNA sequencing analysis and confirmed to be *V. splendidus*. Viable WTVs and MTVs cells were counted after incubation at 28°C for 24 h.

### Statistical analyses

All data are expressed as the mean ± SD of least three sets of independent experiments. Statistical significance was determined by one-way ANOVA with Dunnett’s test. *P* < 0.05 was considered statistically significant.

## Results

### Effect of hppDV.s. on growth, hemolytic activity and anti-UV activity

**Figure 1.**
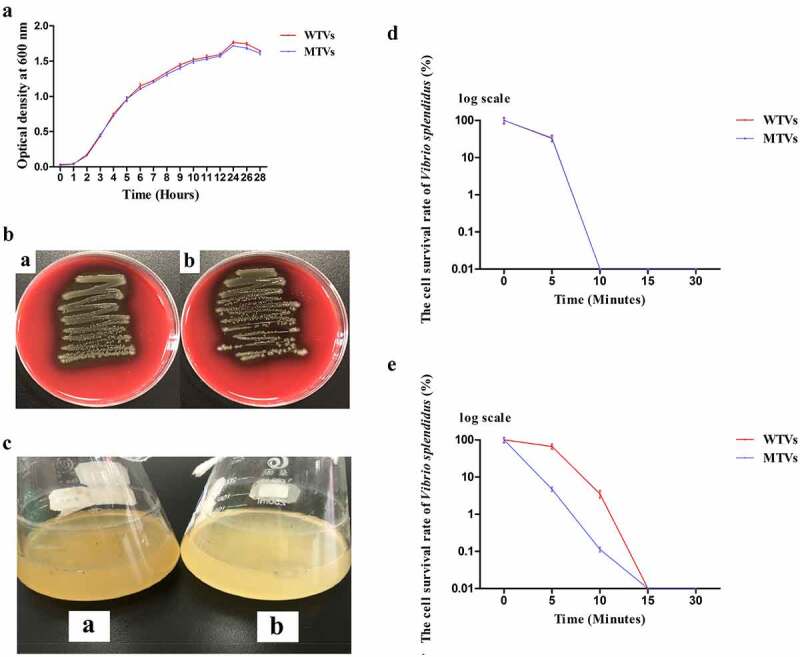
(a) Growth curves of WTVs and MTVs. WTVs and MTVs were spread onto 2216E solid plates at 28°C overnight. Two single colonies were inoculated into flasks with 100 mL of fresh 2216E medium and incubated at 28°C with shaking at 180 rpm. Overnight cultures were diluted to the same concentration, and 200 μL aliquots of WTVs and MTVs were transferred into flasks with 100 mL of fresh 2216E medium. OD_600_ values were measured at different time points. (b) Hemolysis ring on blood agar plate. [a] WTVs and [b] MTVs were spread on 2216E sheep blood agar plate and incubated at 28°C overnight. (c) Colour contrasts of [a] WTVs and [b] MTVs cells cultured in 2216E medium at 28°C until OD_600_ reached 1.5. (d) Bacterial survival rate under UV irradiation (OD_600_ = 0.6). Cell number on solid plates was counted after UV light exposure for 0, 5, 10, 15 and 30 min. The total number of WTVs and MTVs was the same at the beginning, which regarded as 100 and the survival rate was expressed as a percentage. The Y-axes was shown as a log scale. (e) Bacterial survival rate under UV irradiation (OD_600_ = 1.5). Cell number on solid plates was counted after UV light exposure for 0, 5, 10, 15 and 30 min. The total number of WTVs and MTVs was the same at the beginning, which regarded as 100 and the survival rate was expressed as a percentage. The Y-axes was shown as a log scale.

In this study, *hppDV.s*. was knocked out by markerless in-frame deletion to construct MTVs (Supporting Information Fig. S1). The growth rate of the MTVs strain was almost the same as the growth of the WTVs strain (Figure 1a). WTVs showed obvious α-hemolytic activity. MTVs also possessed α-hemolytic activity (Figure 1b). The results showed that the hemolytic activity of WTVs on solid sheep blood plate was similar to that of MTVs. The supernatants of WTVs and MTVs also showed similar hemolytic activities (data not shown). These results suggested that *hppDV.s*. knockout had no obvious impact on the hemolytic activity of *V. splendidus*.

WTVs and MTVs were cultured in 2216E medium at 28°C until OD_600_ reached 0.6 and 1.5 to investigate their anti-UV activity. We observed that the color of WTVs was darker than that of MTVs in the later stages of bacterial growth at the same concentration (Figure 1c). The WTVs and MTVs cells were exposed to UV radiation. The cell counts before exposure to UV light (T = 0 min) were approximately 1.5 × 10^4^ CFU mL^−1^. The cell survival rate of WTVs after UV exposure was similar to that of MTVs at the OD_600_ of 0.6 (Figure 1d). The average number of surviving cells in WTVs were 10^4^, 530 and 0 CFU after exposure to UV for 5, 10 and 15 min, respectively, at the OD_600_ of 1.5. However, the average number of surviving cells in MTVs were 706, 17 and 0 CFU at 5, 10 and 15 min, respectively, at the OD_600_ of 1.5. The cell survival rate of WTVs after UV exposure was higher than that of MTVs at the OD_600_ of 1.5 (Figure 1e). The results showed that hppDV.s. was related to melanin production, which could increase the survival rate and prolong the survival time of bacteria exposed to UV.

### The pathogenicity of MTVs against *A.* *japonicus*

**Figure 2.**
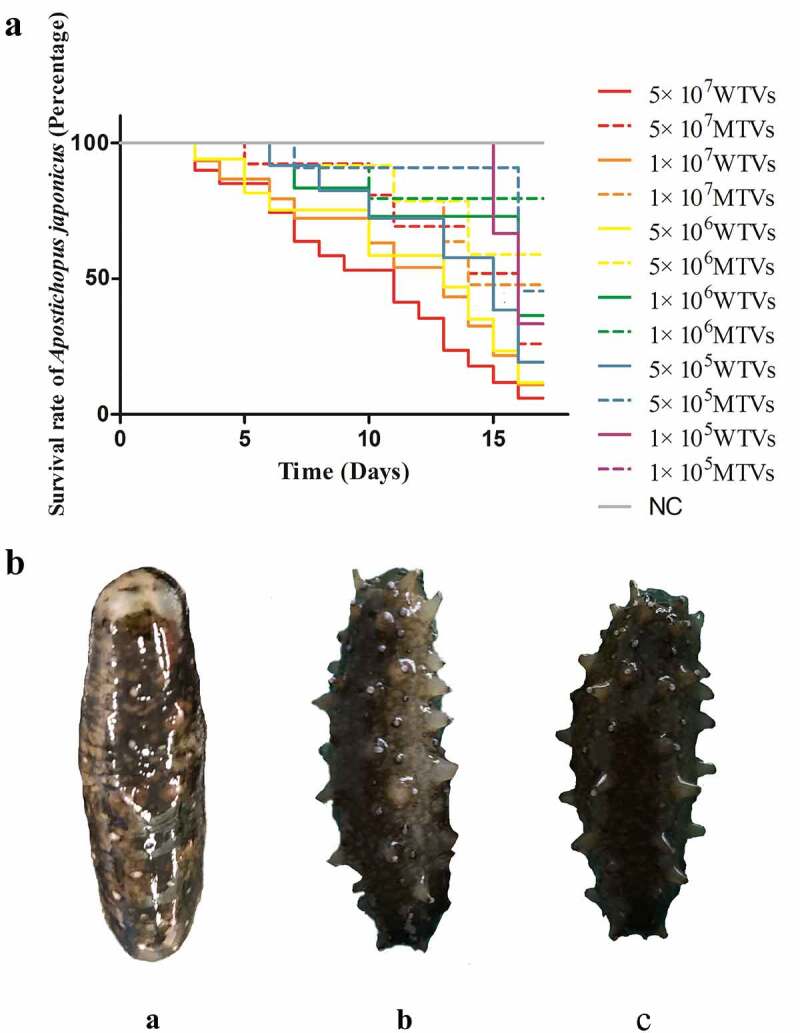
(a) Lethality of WTVs and MTVs. *A. japonicus* was randomly divided into 13 tanks with 20 individuals each. The WTVs and MTVs strains used for infection were cultured in 2216E medium (24 h, 28 °C) until OD_600_ was approximately 1.0. The strains were then washed and re-suspended in PBS (28 °C). For survival assays, weight-matched *A. japonicus* individuals were infected with 5 × 10^7^, 1 × 10^7^, 5 × 10^6^, 1 × 10^6^, 5 × 10^5^ and 1 × 10^5^ CFU mL^−1^*V. splendidus* (WTVs or MTVs). *A. japonicus* infected with PBS was used as the negative control. The water temperature during infection was 16°C. The daily mortality of infected *A. japonicus* was recorded. (b) The observed symptoms of [a] WTVs, [b] MTVs and [c] negative control group (PBS). Dead *A. japonicus* were removed in a timely manner and photographed to observe symptoms.

The *A. japonicus* infected with the MTVs strain exhibited a substantial delay in the time of death and an obvious decrease in mortality compared with those infected with the WTVs strain (Figure 2a). High WTVs concentration led to the acute death of *A. japonicus*. The ﬁrst mortalities of WTVs were observed 3 days post-infection with 5 × 10^7^, 1 × 10^7^ and 5 × 10^6^ CFU mL^−1^ WTVs, and the ﬁrst mortalities of MTVs were observed 5 days post-infection with 5 × 10^7^ CFU mL^−1^ MTVs. The total mortalities of WTVs in 16 days were 17, 10, 10, 4, 6 and 2 post-infection with 5 × 10^7^, 1 × 10^7^, 5 × 10^6^, 1 × 10^6^, 5 × 10^5^ and 1 × 10^5^ CFU mL^−1^ WTVs, respectively. The total mortalities of MTVs in 16 days were 5, 4, 3, 2, 2 and 2 post-infection with 5 × 10^7^, 1 × 10^7^, 5 × 10^6^, 1 × 10^6^, 5 × 10^5^ and 1 × 10^5^ CFU mL^−1^ MTVs, respectively. The *A. japonicus* infected with WTVs exhibited SUS symptoms and a large area of skin rot. However, some less obvious white spots were found on the surface of *A. japonicus* infected with MTVs (Figure 2b). No death or skin rot was observed in the *A. japonicus* individuals in the negative control group. The median lethal doses (LD_50_) of WTVs and MTVs calculated by SPSS were 5.129 × 10^6^ and 2.606 × 10^10^ CFU mL^−1^, respectively.

### Global transcriptional analysis of hppDV.s. in *V.* *splendidus*

**Figure 3.**
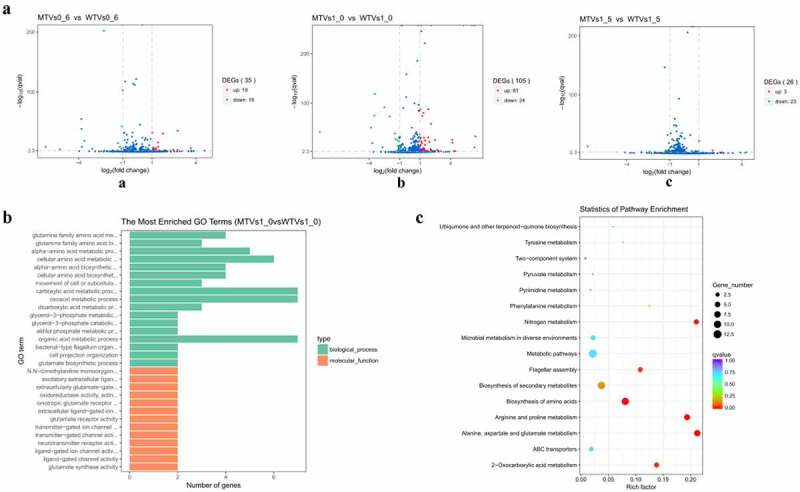
(a) DEG distribution between WTVs and MTVs. Log2 indicates the mean expression level for each gene, and the vertical axis represents the statistical significance of the difference in gene expression. Each dot represents one gene. Red and green dots represent up-regulated and down-regulated DEGs, respectively. Blue dots represent no DEGs. [a] OD_600_ = 0.6; [b] OD_600_ = 1.0; [c] OD_600_ = 1.5. (b) GO enrichment of down-regulated DEGs (OD_600_ = 1.0). (c) KEGG enrichment of down-regulated DEGs (OD_600_ = 1.0).

The raw data were submitted to the NCBI SRA database with accession number SUB10384008. Thirty-five mRNAs from the MTVs exhibited obvious difference compared with those from the WTVs, that is, 16 MTVs mRNAs were down-regulated and 19 MTVs mRNAs were up-regulated in the early log phase at the OD_600_ of 0.6. In the mid log phase at the OD_600_ of 1.0, 105 mRNAs from MTVs exhibited obvious difference compared with those from WTVs; 24 mRNAs were down-regulated and 81 mRNAs were up-regulated. In the stationary phase at the OD_600_ of 1.5, 26 mRNAs from MTVs exhibited obvious difference compared with those from WTVs; 23 mRNAs were down-regulated, and three mRNAs were up-regulated (Figure 3a). The cluster heat map of the differentially expressed genes (DEGs) shows that the expression profiles of genes at the OD_600_ of 1.0 are clearly distinguishable (Supporting Information Fig. S2).

Twenty-four down-regulated DEGs were selected from the OD_600_ = 1.0 group according to log2^fold change^. The top five Gene Ontology (GO) terms were carboxylic acid metabolic process, organic acid metabolic process, oxoacid metabolic process, cellular amino acid metabolic process and alpha-amino acid metabolic process, respectively. The relationships between genes and GO terms are shown in Figure 3b. Bacterial-type flagellum organisation enriched four down-regulated genes, namely, flagellar hook assembly protein (*flgD*), flagellar hook-associated protein, flagellin and flagellar cap protein. According to the Kyoto Encyclopedia of Genes and Genomes (KEGG) database, all 24 down-regulated DEGs were enriched in 16 KEGG pathways (Figure 3c). According to the *P*-value of Fisher’s exact test, metabolic pathways (vsp01100) enriched the largest number of DEGs; the pathway of alanine, aspartate and glutamate metabolism (vsp00250) had the highest ratio; and the pathway of flagellar assembly (vsp02040) was remarkable. These results showed that genes related to metabolic pathways and flagellar assembly were altered in the MTVs strain. Four genes associated with the flagellar system were down-regulated in the MTVs compared with the WTVs; thus, hppDV.s. may regulate the expression of flagellar genes responsible for the swarming motility of *V. splendidus*.

### Amino acid and protein metabolic process

**Figure 4.**
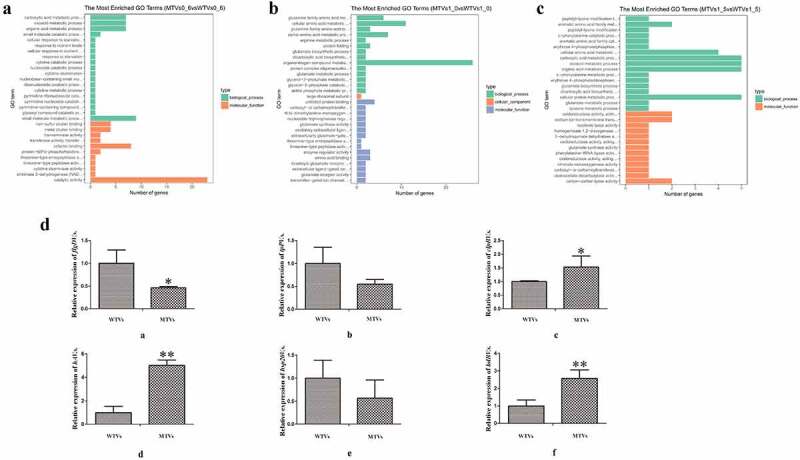
GO enrichment of DEGs. The method used for GO enrichment analysis was GOseq, which can accurately calculate the probability of a GO term enriched by differential genes. The ordinate is the enriched GO term, and the abscissa is the number of differential genes in the term. Green bars represent biological processes, orange bars represent cellular components, and blue bars represent molecular functions. (a) MTVs 0.6 vs WTVs 0.6; (b) MTVs 1.0 vs WTVs 1.0; (c) MTVs 1.5 vs WTVs 1.5. (d) Temporal expression analyses of [a] *flgDV.s*., [b] *tpPV.s*., [c] *clpBV.s*., [d] *hAV.s*., [e] *hsp20V.s*, and [f] *lolBV.s*. in WTVs or MTVs at the OD_600_ of 1.0. Values are presented as mean ± SD (n = 5). Asterisks indicate signiﬁcant differences: *P < 0.05 and **P < 0.01.

In the early log phase at the OD_600_ of 0.6, the GO terms that belong to catalytic activity, small molecule metabolic process and cofactor binding exhibited remarkable difference in MTVs compared with WTVs (Figure 4a). In the mid log phase at the OD_600_ of 1.0, the transcripts involved in amino acid metabolism were substantially down-regulated in MTVs. These genes could encode enzymes involved in glutamate metabolism, which is critical for biofilm formation in numerous bacteria. In addition, ABC transporter-related genes were also considerably down-regulated in MTVs; this down-regulation affected the transportation of nutrients, such as amino acids and sugars, and ultimately affected cell metabolism (Figure 4b). Notably, the structural constituent of the ribosome was also affected by the deletion of *hppDV.s*. in the stationary phase at the OD_600_ of 1.5. The genes that encode 50S ribosomal protein L27, 30S ribosomal protein S9, ribosomal subunit interface protein and 50S ribosomal protein L11 methyltransferase, were down-regulated in MTVs. This result suggests a regulatory mechanism for protein folding and modification process (Figure 4c). In summary, hppDV.s. may regulate bacterial virulence by changing the metabolic process of *V. splendidus*.

### RT-PCR verification of DEGs

The genes selected for the RT-PCR verification of the DEGs at the OD_600_ of 1.0 included *flgD*, ABC transporter permease (*tpP*), heat-shock protein (*hsp20*), ATP-dependent chaperone (*clpB*), outer membrane lipoprotein (*lolB*) and hemagglutinin (*hA*). Melting curve analysis indicated that the RT-PCR product of each gene was unique. The results showed that the RT-PCR results of all genes had a consistent trend with the transcriptome results (Figure 4d). Therefore, the transcriptome sequencing and assembly results had high stability and accuracy.

### Effect on the biofilm formation and swarming motility of *V.* *splendidus*

**Figure 5.**
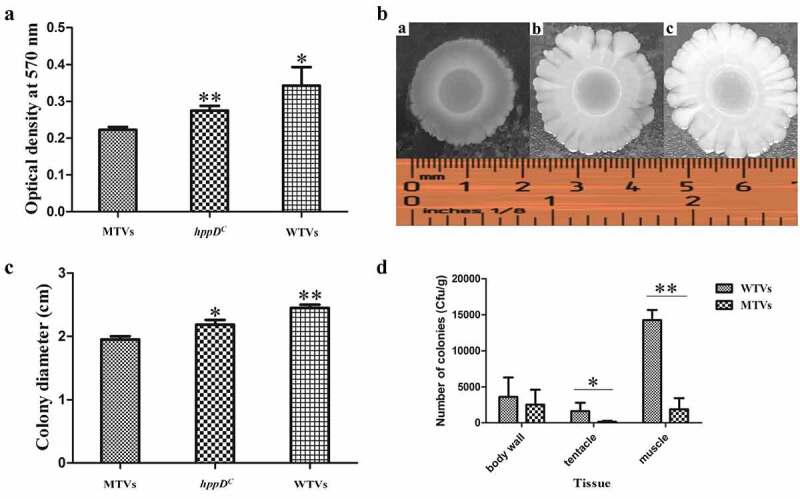
(a) Biofilm formation of WTVs, MTVs and *hppD^C^*. (b) Swimming motility of WTVs, MTVs and *hppD^C^*. [a] The same concentration of MTVs (5 μL) was dropped on low-agar 2216E medium and cultured for 48 h at 28°C. [b] The same concentration of *hppD^C^* (5 μL) was dropped on low-agar 2216E medium and cultured for 48 h at 28°C. [c] The same concentration of the WTVs (5 μL) was dropped on low-agar 2216E medium and cultured for 48 h at 28°C. (c) Bar graph of the swimming motility of WTVs, MTVs and *hppD^C^*. (d) The adhesive ability of WTVs and MTVs to different *A. japonicus* tissues was demonstrated by colony counting. *A. japonicus* was soaked in WTVs and MTVs (1.0 × 10^7^ CFU mL^−1^) for 24 h for infection. The *A. japonicus* tissues were weighed and homogenised. The homogenised solution was diluted gradiently and then coated on 2216E plates. Colony count was determined on the next day. The error line represents the SD (n = 3). Asterisks represent the significance of difference (*P < 0.05, **P < 0.01).

The biofilms of WTVs, MTVs and *hppD^C^* that attached on 96-well plates were analyzed. The results demonstrated that the biofilm formation of MTVs on the surface of a 96-well plate was 81% and 65% of those in *hppD^C^* and WTVs, respectively (Figure 5a). The motility of WTVs, MTVs and *hppD^C^* strains were measured, and the results demonstrated an obvious difference in the swarming motility of WTVs, MTVs and *hppD^C^* (Figure 5(b,c)). The colony diameter of WTVs was 4–6 mm more than that of MTVs at each time point. The diameter of MTVs was 76%–83% of that of WTVs. The swarming motility of MTVs was obviously decreased compared with that of WTVs, and the motility of hppD-supplemented strains was obviously reverted. These results indicated that hppDV.s. positively regulated the swarming motility of *V. splendidus*.

### Distribution of WTVs and MTVs in various *A.* *japonicus* tissues

The quantitative detection of cell adhesion in various tissues of *A. japonicus* after immersion infection with WTVs and MTVs is shown in Figure 5d. The adhesion quantities of WTVs to the body wall, tentacle and muscle tissues were 3.87 × 10^3^, 2.54 × 10^3^ and 1.39 × 10^4^ CFU g^−1^, respectively. The adhesion quantities of MTVs to all tissues except the body wall obviously decreased. The adhesion quantities of MTVs to the body wall, tentacle and muscle tissues were 3.16 × 10^3^, 3.62 × 10^2^ and 2.97 × 10^3^ CFU g^−1^, respectively.

## Discussion

The toxicity of many pathogens depends on different virulence factors [33]. Many genes have been demonstrated to be involved in the virulence regulation of aquatic pathogenic bacteria by knockout technology [^34–37^]. However, no studies on the contribution of the knockout of the *hppDV.s*. gene to the pathogenesis of *V. splendidus* have been reported. The 4-hppD catalytic reaction is the ﬁrst committed reaction of the tyrosine catabolism pathway in essentially all aerobic organisms [38]. Homologues of 4-hppD have been identified from the bacterial pathogens *Streptomyces avermitilis* [39,40], *Vibrio vulniﬁcus* [41], *V. splendidus* [17], *Pseudomonas ﬂuorescens* [42] and *Legionella pneumophila* [43,44]. In our previous studies, the dual functions of 4-hppD for *V. splendidus* cytotoxicity and survival was determined by in vitro experiments [22]. However, the roles of 4-hppD in the pathogenesis processes of *V. splendidus* on *A. japonicus* are rarely reported. Thus, we launched a series of experiments to determine how the *hppDV.s*. gene influences the virulence of *V. splendidus*.

Our previous research demonstrated that hppDV.s. contributes to the α-hemolytic activity of *V. splendidus* [17]. However, the present result showed that *hppDV.s*. knockout had no substantial effect on the hemolytic activity of *V. splendidus*. The result demonstrated that the hemolytic activity of *hppDV.s*. was overlapped by other hemolysins. The mechanism of the hemolytic activity of *V. splendidus* is complicated. Hemolysins from *Vibrio* sp. have many types [1]. We also found other kinds of hemolysins, such as thermolabile hemolysin and hemolysin III family protein [45], in the *V. splendidus* genome. This finding means that despite *hppDV.s*. was knockout, alternative pathways recompense the hemolytic activity of *V. splendidus*. Furthermore, hppDV.s. produces pyomelanin from tyrosine via HGA [22]. A study reported that the melanin production of mutant *Bacillus thuringiensis* is more resistive to UV radiation than that of the wild strain [46]. In the present study, we observed that the color of WTVs was darker than that of MTVs at the same concentration as time increased, and the survival rate of MTVs exposed to UV was significantly decreased compared with that of WTVs in the later growth stages. As depicted in Supporting Information, the expression of *hppDV.s*. reached the highest level at the OD_600_ of 1.6 and reverted to the original level at the OD_600_ of 2.0 (Fig. S3). Levin et al. also found that *L. pneumophila* secretes HGA after the bacteria stops replicating during stationary phase [47]. Their finding is consistent with our findings. The results suggest that *hppDV.s*. is expressed during the stationary phase and secrete HGA to participate in self-protection.

The *A. japonicus* infected with the MTVs strain exhibited a substantial delay in death, a remarkable decrease in mortality and alleviated SUS symptoms compared with those infected with the WTVs strain. Furthermore, the LD_50_ was reported for the ﬁrst time to show the direct pathogenicity of *V. splendidus* to *A. japonicus*. These results suggested that the knockout of *hppDV.s*. led to the decrease in the virulence of *V. splendidus*. We collected WTVs and MTVs cells at the OD_600_ of 0.6, 1.0 and 1.5 for bacterium RNA-seq to explore the changes in the bacterial virulence of MTVs. The OD_600_ of 0.6 and 1.5 are the early log phase and stationary phase of *V. splendidus*, respectively. The mRNAs from MTVs exhibited less substantial difference compared with those from WTVs during these stages. However, in the mid log phase at the OD_600_ of 1.0, 105 mRNAs from MTVs exhibited remarkable difference compared with those from WTVs; thus, these genes may be associated with the pathogenicity of *V. splendidus.*

hppDV.s. regulates cell metabolism through enzyme activity. HGA generates fumarate through a series of reactions, and fumarate can participate in the TCA cycle, which is the pivot of substance metabolism (Fig. S4). α-Ketoglutarate is the carbon skeleton of glutamate and is derived from the TCA cycle. We found that the expression level of glutamate synthase remarkably decreased in MTVs according to the transcriptome data. A glutamate synthase mutant exhibited an early arrest of biofilm formation [48]. The bacterium manages to persist in the environment by associating with biofilms, which are microbial communities that attach to surfaces and encase themselves in a protective matrix [49,50]. In addition, previous research suggested that the high-density colonies of *Legionella* protect themselves by secreting HGA pulses, and biofilm formation is accompanied with pyomelanin production in deep-sea *Pseudoalteromonas* sp. SM9913 [47]. These results indicated that hppDV.s. is closely related to biofilm formation. However, no research has shown how the tyrosine metabolism mediated by hppDV.s. affects biofilm formation. The current research demonstrated that tyrosine metabolic disorder reduces a metabolic intermediate that prevents the system from regulating biofilm formation. These findings increase our understanding of the coordination between cellular metabolic status and the regulatory networks that govern biofilm formation. The results of GO and KEGG analyses showed that the GO term “bacterial-type flagellum organisation” and the flagellar assembly signaling pathway were considerably down-regulated by *hppDV.s*. knockdown. We speculated that flagellar assembly cannot proceed normally because of the decreased TCA cycle flux, which causes the down-regulation of flagellar biosynthesis genes [51]. A previous study found that flagellar systems are responsible for motility and are important for the infection and colonisation of *Vibrio coralliilyticus* and *Vibrio parahaemolyticus* [52,53]. Given the importance of the complex interaction relationship between biofilm formation and flagellar motility as depicted in Supporting Information Fig. S5, *hppDV.s*. knockdown may change the dispersion of *V. splendidus*. The results also demonstrated that the MTVs strain was less virulent and less abundant in *A. japonicus* with lower swarming motility.

HGA is the precursor to melanin production in bacteria and plastoquinone production in plants [54,55]. In addition, the emergence of acetyl-CoA and histone acetylation could influence pentose phosphate pathway (PPP) flux and cause a series of changes in mammal cells [56]. Our previous study demonstrated that hppDV.s. exhibits cytotoxicity to the coelomocyte of *A. japonicus*, and purified rhppDV.s. can cause oxidative stress in *A. japonicus* [22]. Excessive ROS levels can cause cell damage [57,58]. Interestingly, we also demonstrated that *A. japonicus* 4-hppD could down-regulate ROS levels through the 4-hppD–glucose-6-phosphate dehydrogenase–PPP pathway within the normal range [59]. Moreover, the transcriptome of *A. japonicus* stimulated by WTVs and MTVs revealed that the p53 and NOD-like signaling pathways are involved in the resistance to the pathogenicity regulated by hppDV.s. (data not shown). Combined with the results of this article, we revealed the different functions of the *hppD* gene between hosts and pathogens (Fig. S6).

In conclusion, the deletion of the *hppDV.s*. gene in *V. splendidus* showed no effect on *A. japonicus* growth, but MTVs infection resulted delayed onset time, reduced mortality and alleviated SUS symptom in *A. japonicus*. RNA-seq indicated that glutamate metabolic pathways and flagellar assembly genes were altered in the MTVs strain. hppDV.s. regulates biofilm formation and flagellar assembly, which are responsible for the virulence of *V. splendidus*, by changing metabolic processes. These ﬁndings provide a theoretical basis for the research of disease prevention and control.

## Supplementary Material

Supplemental Material

## Data Availability

The data that support the findings of this study are openly available in [GenBank SRA database] at https://www.ncbi.nlm.nih.gov/bioproject/?term=PRJNA763740, reference number [PRJNA763740]. Other data supporting the findings of this study are available within the article and its supplementary materials.
