# Supplementary material for: Vibrio splendidus virulence to Apostichopus japonicus is mediated by hppD through glutamate metabolism and flagellum assembly
Source: Virulence. 2022 Mar 8;13(1):458–70. doi: 10.1080/21505594.2022.2046949 (PMC8920201; doi:10.1080/21505594.2022.2046949)
Supplement: Supplemental Material [file KVIR_A_2046949_SM5156.docx]

**
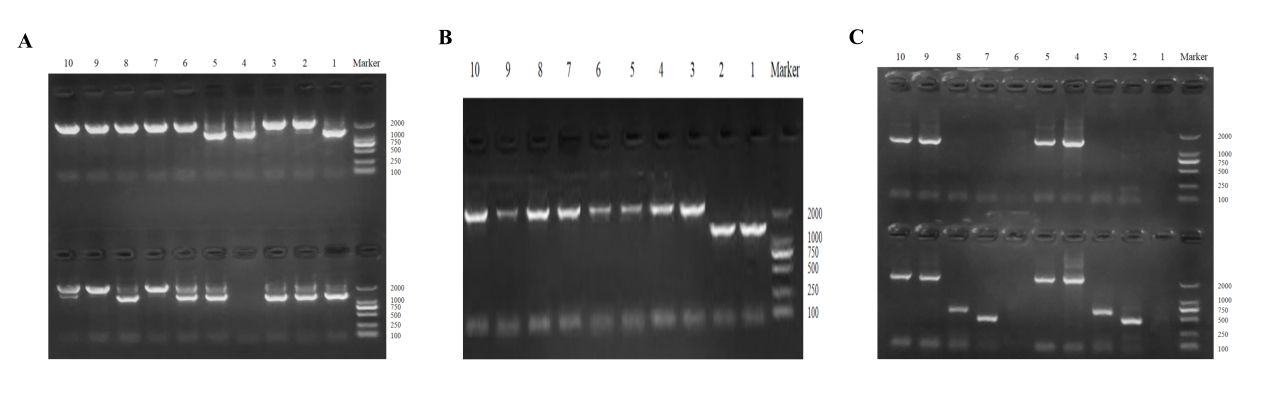
**

**Fig. S1** (A) First homologous recombination. Vs has no π gene. pDM4Ko*hppDV.s.* must be recombined and exchanged to Vs DNA to replicate, and appropriate concentrations of Cm and Amp resistance provide selective pressure. Two bands were detected by KohppDV.s.F1/KohppDV.s.R2. The long 2426 bp band is the sequence on the Vs genome, and the short 1376 bp band is the sequence on the pDM4Ko*hppDV.s.* plasmid. (B) Second homologous recombination spread on solid 2216E (12% sucrose, Amp) plates and cultured overnight at 28 °C. Single colonies were picked and tested with KohppDV.s.F1/KohppDV.s.R2 primers, and the single colonies with a short band (1376 bp) were selected. (C) WTVs and MTVs verification. Lanes 2–5 in the upper row are the MTVs 1 produced after PCR, lanes 7–10 in the upper row are the MTVs 2 produced after PCR, lanes 2–5 in the lower row are the WTVs 1 produced after PCR, and lanes 7–10 in the lower row are the WTVs 2 produced after PCR. Lanes 2, 3, 7 and 8 are the products of PCR primers VsGly1F/VsGly1R and VsGly2F/VsGly2R, which amplified the *hppDV.s.* domain. Lanes 4, 5, 9 and 10 are the products of PCR primers KohppDV.s.F1/KohppDV.s.R2.

**
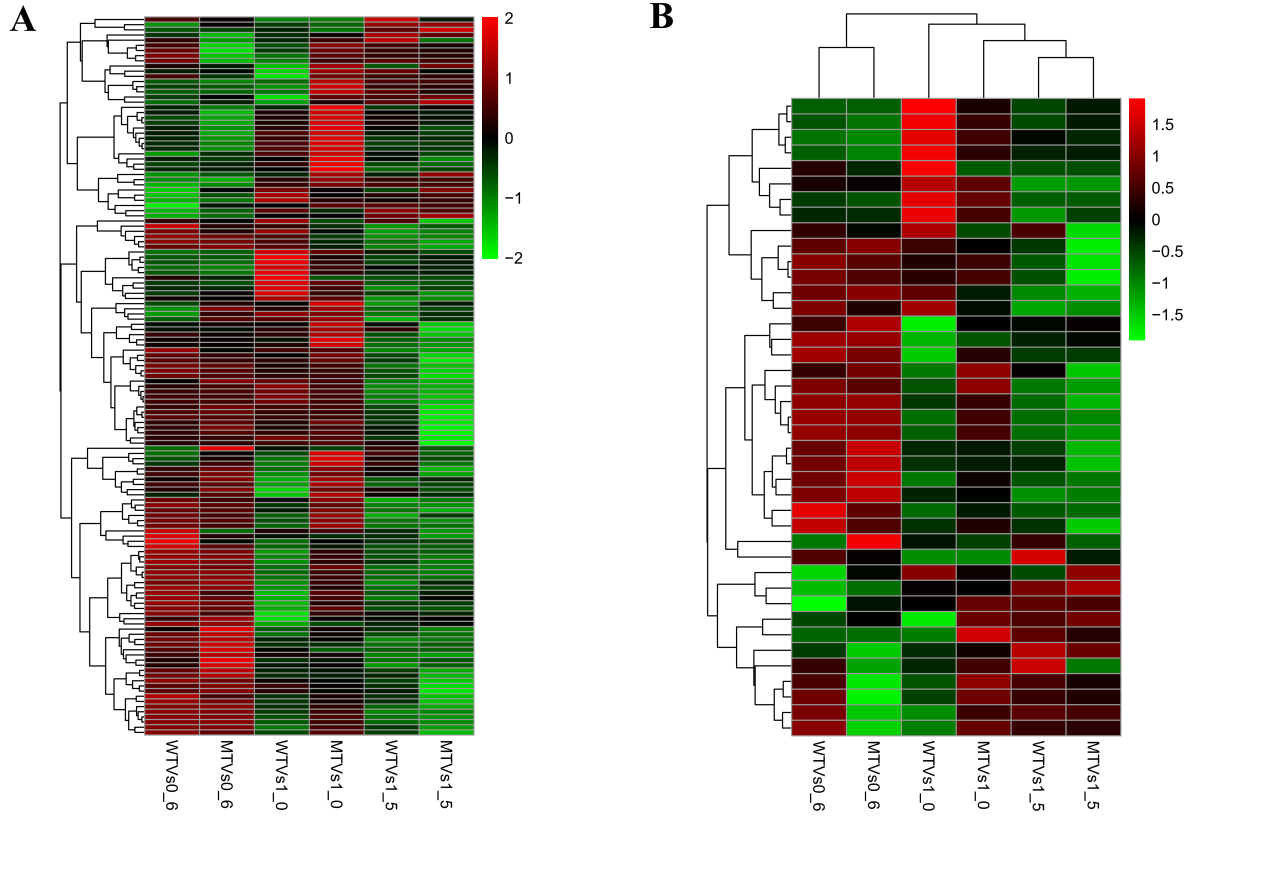
**

**Fig. S2** Hierarchical clustering of (A) all DEGs and (B) DEGs in [metabolic pathways](F:/学习/细菌敲除转录组/X101SC19061578-Z01-F001-B1-16_TR_result/X101SC19061578-Z01-F001-B1-16_results/8.DEG_KEGGEnrichment/8.3.DEG_KEGGPath/ALL/MTVs1_0vsWTVs1_0/src/vsp01100.html). For hierarchical clustering, green and red indicate depleted and enriched expression, respectively. Transcripts were clustered by hierarchical clustering using the complete linkage algorithm.

**
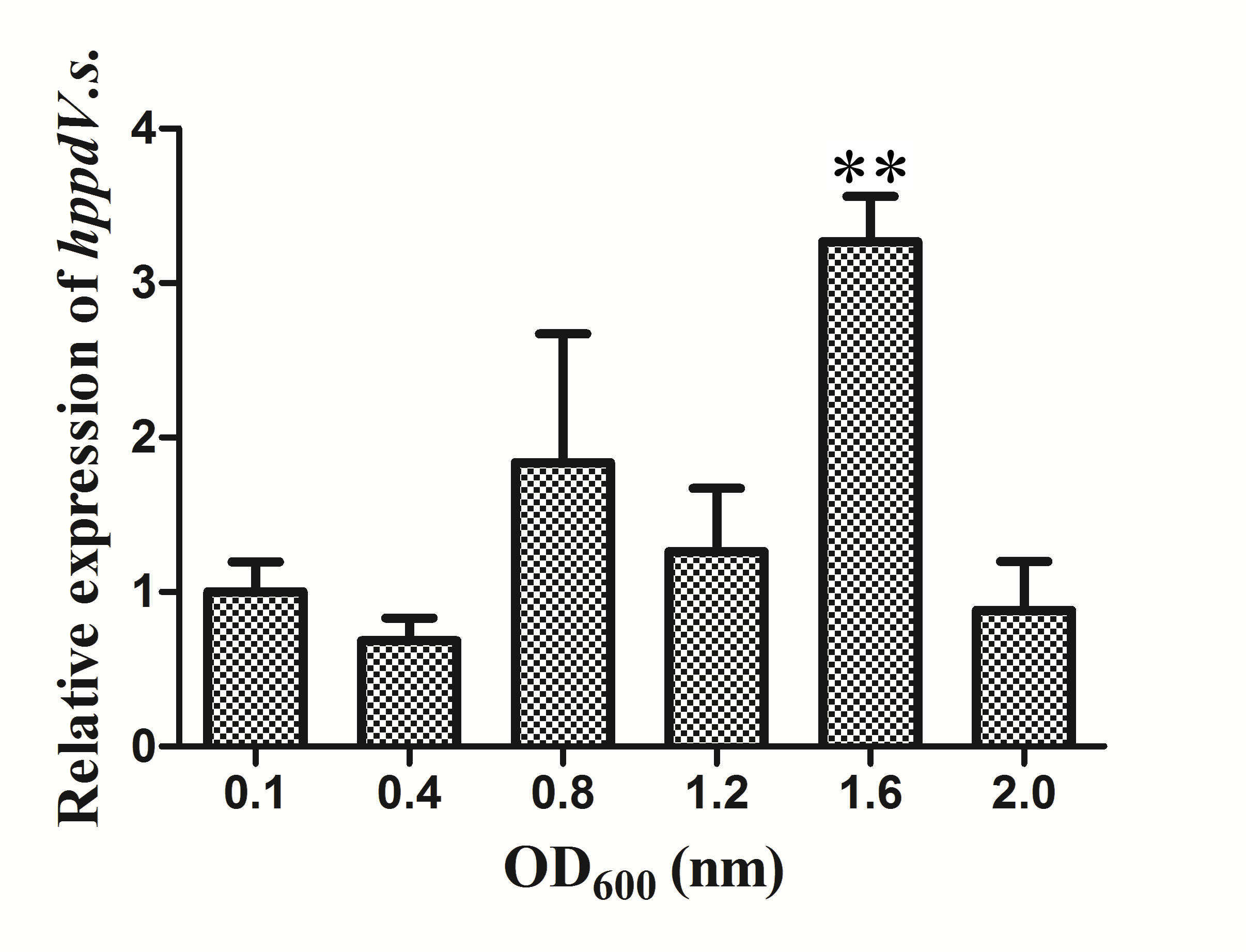
**

**Fig. S3** Expression levels of *hppDV.s.* in different bacterial concentrations. Data are the means ± SD of three independent experiments. * *P* < 0.05, ***P* < 0.01.

**
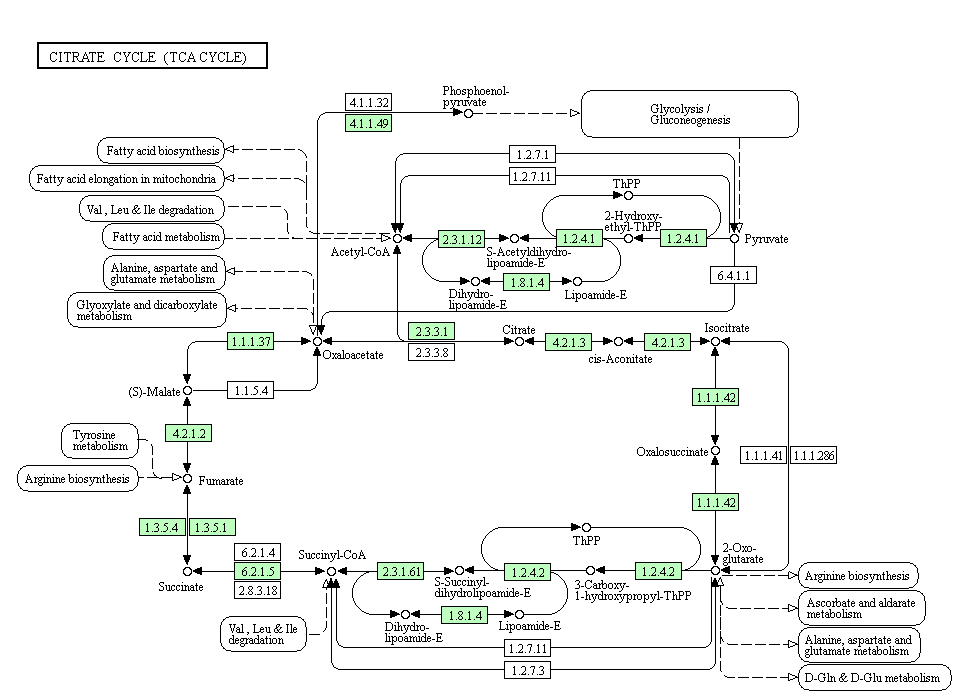
**

**Fig. S4** KEGG pathway of the TCA cycle.

**
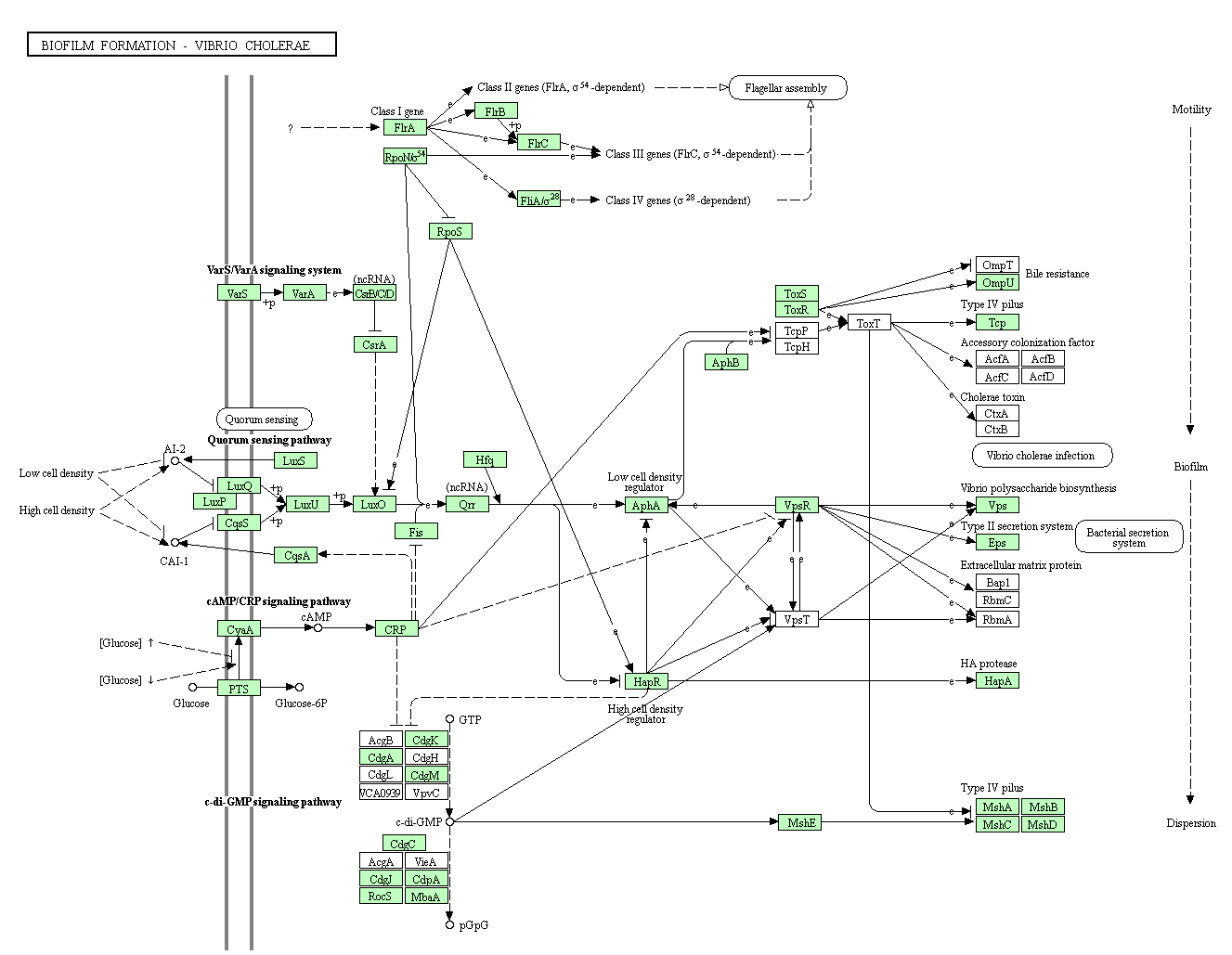
**

**Fig. S5** Motility, biofilm formation and dispersion according to *V. cholerae* biofilm formation mechanism

**
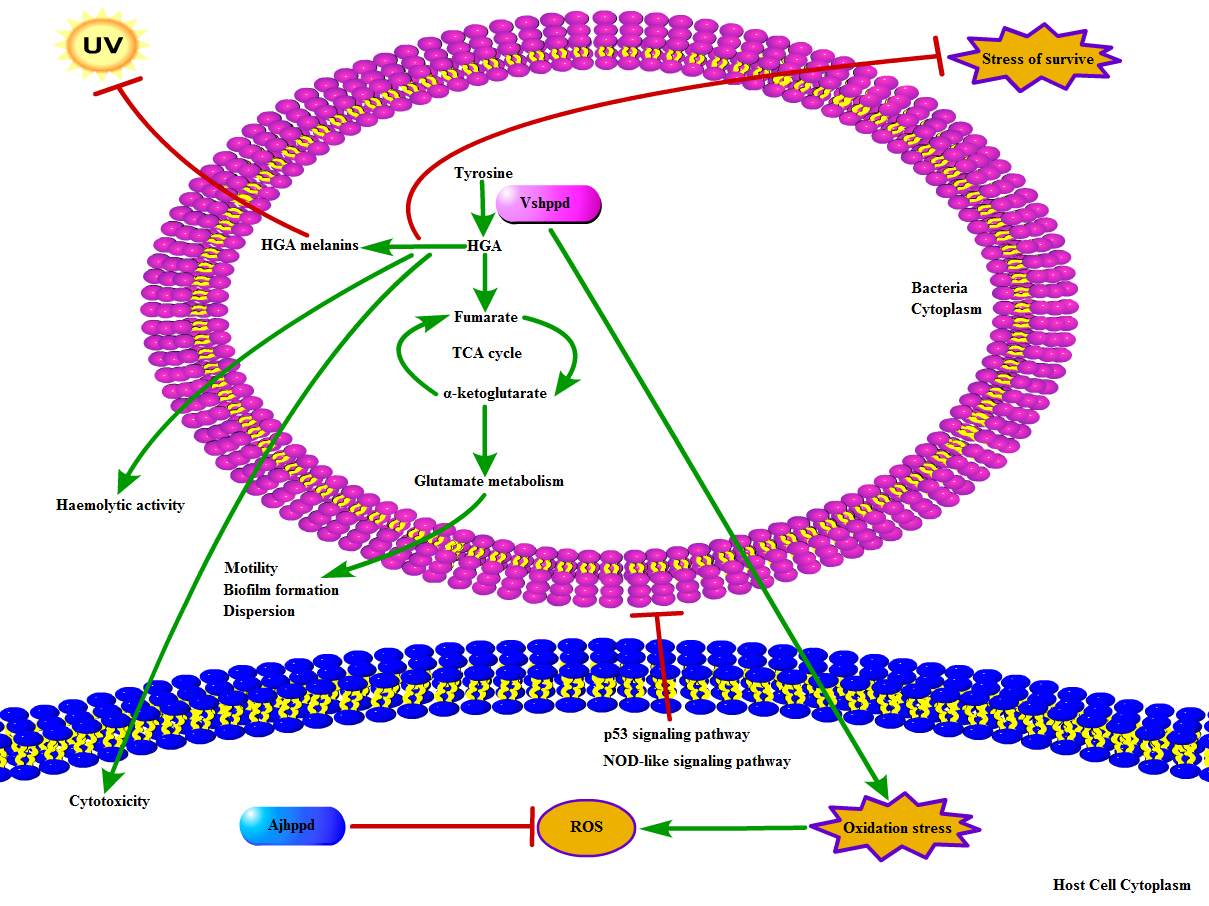
**

**Fig. S6** Working model of how *hppDV.s.* controls metabolic and virulence-related processes in *V. splendidus*. Arrowed and bar-ended lines indicate activation and repression, respectively.
